# On the Treatment of Fever by Cold Water

**Published:** 1848-02

**Authors:** William Gill

**Affiliations:** Physician to the Nottingham Dispensary, &c.


2. *On the Treatment of Fever by Cold Water.* By William Gill, M. D., Physician to the Nottingham Dispensary, &c. (Provincial Medical and Surgical Journal, September 22.)

[In our last volume, p. 3, the reader will find a communication by Mr. Stallard, of Leicester, upon the efficacy of the external application of cold water as a refrigerant and sudorific in fever; we continue the subject by the following abstract of a paper which was read at the last meeting of the Provincial Medical and Surgical Association.]

"Before entering more immediately on the object of this paper, the author describes concisely the general features of the prevalent fever. In most cases the *immediate* cause of the attack was traceable to sleeping in crowded lodging-houses, the usual abode of fever in large cities; the proximate causes, doubtless, were over-fatigue, and insufficient and unwholesome food. The term "hunger pestilence" has been aptly applied to the disease. A true typhoid gastro-enterite was present in many of the patients, closely resembling what so frequently is observed in the Parisian hospitals. Whether the essentiality of the fever existed in the condition of the muco-alimentary membrane or not, it was not the author's intention to discuss. This, however, he remarked, that *so soon* as the signs of gastro-alimentary irritation were subdued, the signs of general fever subsided. Some two or three cases, which he read, corroborate this observation. In the generality of patients under his care, not only was the gastro-alimentary membrane affected, but also the muco-pulmonary, as evinced by cough, shortness of respiration, and frequently universal sonorous râles, affecting the whole of the chest. In most of the Irish sick, the skin was spotted with petechiæ, of different sizes and color, chiefly developed on the abdomen and chest. This was not remarked amongst the English cases. There was no discharge of blood from the inner membranes. (Edema of the lower extremeties occurring early in the disease was generally a fatal symptom, though we had two cases of recovery in boys, who were universally anasarcous from the commencement. The disturbance of the sensorium was marked by a low muttering delirium, sometimes wandering about the bedroom, constant picking at the bedclothes, and subsultus tendinum. Some were affected with a heavy, comatose, and stupid state, from which they were with difficulty aroused, and when aroused, with difficulty were made to understand questions; they relapsed immediately into the same lethargic condition when left to themselves. This comatose condition often continued till convalescence was established, and in some even later. It seemed a perfect prostration of all mental energy, and was only relieved as the bodily powers regained their tone. In no one case did active delirium occur. The secretions from the bowels were thin, frequent, black, and offensive, and often attended with severe griping, but no bloody dicharges. The function of the bladder in one or two individuals was suspended, and it was necessary twice daily to use the catheter. The usual period of the termination ot the fever seemed to be from the eighteenth to the twenty-first day, at which time the patients were left in a state of the greatest prostration. When the case terminated fatally, an unrousable, unconscious coma closed the scene. The usual symptoms of fever were generally present,—as the hot dry skin, black tongue, urgent thirst, pulse varying from 90 to 130, insomnia, and pains in head, back, and limbs, &c. After this brief description of the general features of the disease, he proceeds to the treatment.

He remarks that he is well aware that a great prejudice exists in the profession against the treatment to be advocated, partly because it is opposed to preconceived opinions, and chiefly from the unprofessional manner in which it has been ushered into notice Feeling certain, however, that he was addressing a body of gentlemen willing to rereceive *truth* for the *sake of itself*, he, with perfect confidence, detailed a treatment of fever as yet untaught in the schools, and generally unrecognized by the profession.

Dr. Currie, of Liverpool, was the first scientific English physician who enlisted cold water as an external remedial agent in the treatment of fevers. Successful as the practice was under his direction, it has been little followed in later times. It is only within the last few years that the prejudices which existed against the internal and external use of water has begun to subside. "Perhaps," observes the author, "the prominence of the sanatary questions, and the many evils proved to arise from the want of a due supply of pure water, has had much to do in removing this groundless prejudice, and may have produced an undue reaction in its favor, causing it to be considered *not only* as necessary to a healthy condition, but as a *curative agent* of universal efficacy. Hence, perhaps, the public mind has been somewhat prepared to receive the hydropathic theory with much more favor than its intrinsic merits demand.—An universal remedy will ever find many advocates, and in a numerous profession like ours, there are ever men to be found who, from selfish motives, will pander to this diseased taste of the public mind. We as an association, must ever protest against such exclusive theories as prevail in our days, being in our opinion unscientific, opposed to experience, and calculated to lead to incorrect views respecting the power of many known and valued medicinal agents. In making this protest against any exclusive theory for the cure of diseases, we must not rush into the opposite extreme, and, from disbelief of their universal efficacy, deny their particular efficacy, when the touchstone of experience speaks to the contrary."

The plan the author has adopted for the cure of fever, has been a modification of Dr. Currie's. Instead of pouring buckets of cold water over the body, he has it enveloped in a wetted sheet, an instrument more effective than Currie's in respiration, which did not uniformly follow his plan. The fear of evil consequences from the treatment is groundless. He gives no opinion as to its utility, except in cases of fever. Here, however, he states that he can speak with confidence. When the skin is burning hot, and the mouth and tongue parched, the application of a sheet wrung out of cold water, and applied *closely* to the whole surface of the body, and evaporation prevented by the application of three or four blankets placed over it, produces a most grateful feeling of refreshment, which is soon followed by a more or less warm perspiration. In young people, this perspiration breaks out in from five to ten minutes after its application; in middle-aged people the period is longer. Many uncomfortable sensations are soon relieved by its use; such as muscular pains in the back, thighs and legs, and the sense of aching and weariness; the thirst often becomes less, and even the dry tongue sympathises with the relaxing influence induced on the cutaneous surface. He has seen the low moaning delirium subside whilst under its use; and some patients, who have not slept before, doze, especially if the hair has previously been cut short, and a flannel nightcap, wetted with vinegar and water, been applied to the head.

The simple plan he has followed has been this:—On a flock-bed he has placed from three to five blankets; superimposed over these, a sheet wrung out of cold water, on which the patient, stripped, is placed, with legs outstretched, and arms to the side; the sheet is then drawn tightly around, up to the neck, and inclosing the feet; first one blanket, then another, and so on to the whole number, are tightly drawn over the sheet, so as to have the *whole body well and closely packed.* In this state, the patient lies from a quarter of an hour to one or two hours, according to the object in view, and the effect produced. Some get tired at the end of half an hour, some can continue for one or two hours, and feel very comfortable. As soon as a gentle perspiration commences, a wineglassfull of water is given frequently. At the commencement of this treatment, in a case of fever, he has generally ordered its use for one hour; after that time the wet things are removed, and the sick person is placed in bed, well wrapped in three blankets, and allowed to perspire for three hours; afterwards the blankets are to be carefully removed, one at a time, so as to allow the perspiration to subside gradually, and the patient is then placed in bed, between the sheets.

During the whole of this period, small quantities of water should be given. In the summer, during this process, a free ventilation may be allowed in the chamber, in winter it is necessary to have a good fire, and to have one blanket well warmed, to apply around the body, so soon as removed from the wet sheet.

Several cases of incipient fever have lost all traces of disease after the first application. If the fever be not reduced, the next day the same plan must be repeated, keeping the patient in the wetted sheet from half an hour to one hour, according to the intensity of the symptoms, and in the blankets from one to two hours. This may be repeated every day till indications of *a cool* skin arise, then it must be immediately discontinued.

During some period of this treatment, the temperature of the atmosphere being very high, (75° to 78° in shade,) the author has not found it advisable to keep the patient as long as two hours sweating in the blankets; from an hour to one hour was sufficient. A longer period caused the pulse to be accelerated instead of lowered, which latter is the usual effect of the treatment. In very hot weather, when a free perspiration has been induced at the commencement of the fever, he has adopted the following plan. To wrap the sick person for half an hour in the wet sheet, covered lightly with one blanket; to be then washed all over with a towel wetted in tipid water, then rubbed dry, and placed in bed between the sheets. He has not found it necessary to make use of this treatment more than five times to the same individual; generally after the third or fourth application, the skin becomes cooler, and the other signs of the fever gradually subside. When the skin becomes cool, and the tongue less dry, he has instantly discontinued all water remedies, and given bark, wine and broths, and it was surprising how soon convalesence and strength became established. During the whole course of the fever, milk and water, or weak broths, were allowed *ad libitum.* In one person, twice in the course of the same day, owing to the intensity of the fever, it was found necessary to repeat the wet sheet, using it the second time for only half the period of the first; a comfortable night ensued.

Without doubt, this is a most effective mode of *quickly* reducing the temperature of the body; an equilibrium is soon established between the cold of the water and the heat of the body, and the patient becomes bathed in a natural vapour-bath, as may be felt by placing the hand under the bedclothes. Where the fever runs high, and the delirium is violent, the wet sheet may be safely applied for short periods (two minutes,) several times in the course of the day. This will be found a more effectual mode of reducing the cerebral excitement than any other means with which we are acquainted. This refrigerating plan, used for ten minutes, during an evening exacerbation, will often produce a few hours' refreshing sleep.

The author confesses that he had, at first great doubts as to the *safety* of this treatment, where the mucous membranes of the bronchi and gastro-alimentary passages were complicated. Very soon his fears on this head were dissipated by the convincing evidence of experience; in fact, *these* proved the case in which the decided benefit of the treatment was most marked. The quick and embarrassed respiration, dry cough, and sonorous râles, subsided quickly after one or two applications of the wet sheet; the cough became looser, the râles moister, and expectoration was established.

The same happy change also occurred where the gastro-alimentary membraneswere disordered. Generally, the first wet sheets puts a stop to the diarrhœa, and soon afterwards, pain and swelling disappeared. A confined state of the bowels was frequently the effect of the wet sheet, and it was found necessary, in several of the patients, to resort to small doses of castor oil. In three or four cases, the symptoms of gastric and abdominal irritation or inflammation were so violent as to have justified the employment of leeches in the usual treatment followed in the Parisian hospitals, and yet by the simple means mentioned, in three days every bad symptom had vanished. A great saving is made to the patient's strength, when we can dispense with the abstraction of blood.

As the author is anxious to make this paper altogether practical, he does not enter into any theory respecting the *modus operandi* of the wet sheet.

The following selection of cases was read:

CASE I. Michael Kane, aged 18, Irish vagrant, of vigorous constitution. He has been in the Union Hospital five days, under the care of Mr. Stiff, and taken salines.

June 28th. The following is his present condition:—Supination in a lethargic state, and unconscious, unless violently aroused; the face purplish red; eyes bloodshot and pupil dilated; constant picking at the bedclothes; subsultus tendinum; low muttering delirium; the skin furnace-hot; tongue dry, shrivelled, black, and covered with sordes; diarrhœa; general tympanitis of abdomen, with much expression of pain when pressed, unless aroused, and then his face indicates the existence of pain; the urine and stool are not passed involuntarily; the abdomen and skin generally covered with dark-colored petechiæ; the respiration hurried, forty-four in the minute, and the stethoscope reveals universal bronchitic râles in the chest; pulse 130, weak and hurried. The treatment ordered was the application of the wetted sheet for one hour, blanket for two hours; the head to be shaved, and a flannel night-cap, wetted with vinegar and water, to be constantly applied. To have milk and water *ad libitum*.

There evidently were clear signs of head, chest and abdomen complication. The bloodshot eye and purple countenance, accompanying a nearly unconscious state, indicated a congestive condition of the brain. The stethoscope revealed a similar condition in the lungs, and the universal swelling of the abdomen, attended by diarrhœa, and by pain when the patient was partly sensible, added no little to cause a most unfavorable prognosis to be formed.

June 29th. The aspect is better; has passed a better night; the picking at the bedclothes and the low muttering delirium are quite subsided; the skin is cooler and rather inclined to moisture; the purging no longer continues, and there is less tympanitis; breathing and dry cough less troublesome; respiration not so frequent when lying quiet but the slightest movement causes it to be accelerated; the râles moister; the man more intelligent when aroused, but still instantly falls into a doze when left to himself; the tongue not so black or dry ; the pulse come down to 100, regular and soft. He sweated much both in the sheet and blankets. To repeat the wet sheet and blankets as before.

30th. Continues better in all respects. No further application of the wet sheet.

July 1st. The man is convalescent; skin cool and moist; tongue has nearly lost all marks of dryness and blackness; urine free and paler colored ; bowels open once daily ; intelligence nearly restored ; pulse 90 ; the chest and abdominal complications rapidly subsiding ; the patient asks for nourishing diet. To have the bark, mutton broth, and bread and milk.

July 4th. To have meat daily.

5th. Is able to walk in the room.

6th. Is down stairs in the yard, and well.

Case II. Martin Glynn, Irish vagrant, aged 13, has been ill three days.

June 9th. There is intense heat of skin, and flushing of the face, with pains in the head, bones, abdomen, back, and legs ; great thirst ; tongue deep red and covered in the centre with a cream-colored fur; great pain in epigastrium, and a tympanitic condition of the abdomen, with diarrhœa ; there exists slight cough, but no râles in the chest ; the tongue is tremulous and subsultus tendinum is present ; no sleep; pulse 110, rather sharp ; urine scanty, and high colored.

To have the wet sheet for one hour, and blankets for three hours. Milk and water to drink. The abdominal complication was most marked in this case—*a true typhoid gastro-enterite.*

10th. Continues in many respects the same ; the diarrhœa, however, has subsided. Was ordered a repetition of the treatment, and the vinegar and water lotion to the head.

11th. Says he is better to-day ; the skin is cooler, and inclined to moisture ; face very little flushed ; tongue becoming less dry and red ; headache better ; no pain in epigastrium or abdomen ; bowels confined ; urine free and paler; less thirst; pulse 110, but not so sharp. To repeat the wet sheet as before.

12th. Convalescent ; slept the whole of the night, and makes no complaint this morning, except weakness. Face cool ; headache gone ; tongue clean and moist ; urine free; pulse 64, very soft; appetite returning. To have mutton broth and bread and milk.

13th. To have rice pudding and meat.

16th. Is able to walk in the yard, and may be considered well.

Case III. Thomas Gafen, Irish, aged 14, of healthy habits, ill for three days.

July 8th. Face flushed and anxious; skin very dry and hot; tongue of a vivid red, and in the centre covered with a dirty cream-colored fur, becoming dry and black in places; great thirst; throbbing pain in the head, epigastrium and limbs; pulse 120, wiry, and small; considerable tenderness in epigastrium; gurgling in iliac region, accompanied with diarrhœa; the respiration hurried; frequent cough, and universal sonorous râles in the chest; no sleep, urine scanty. Was ordered the wetted sheet for one hour, the blankets for two hours. Hair to be cut short, and the wetted cap applied. Milk and water to drink.

9th. Continues in all respects the same, except that the skin is somewhat cooler.

10th. Wonderfully better; slept much in the night; aspect natural; no heat of face or skin, which is inclined to moisture; tongue moist, and losing its fur; very slight thirst; urine free; bowels open twice since the 10th, and has lost all pains in the head and epigastrium; pulse 76, soft. No further application was ordered.

On the 14th the boy was allowed to sit up, and have meat, and was considered convalescent.

In conclusion, the author inquires whether we may not draw the following conclusion from the facts brought forward:

1. That the judicious use of the wet sheet has a powerful influence in relieving many of the most distressing symptoms of fever.

2. That if applied *very early* in the disease, it may in some cases arrest its further progress.

3. That if used *later* in the disease it has a controlling influence, bringing the fever to a termination much earlier than by any other known treatment.

4. That the ordinary *complications* of fever are no arguments against, but rather for its use.

5. That with this treatment, weak broths and milk and water, *ad libitum*, may be allowed.

6. That the first symptoms of the subsidence of the fever, were a cool and often moist condition of the skin, a diminution of thirst, and an improvement of the tongue.—When these changes occur, the treatment must directly be discontinued, and the bark and better diet be ordered.

7. That some of the worst cases of typhus fever were convalescent, and walked about on the fifteenth day from the commencement of the attack.

[We may further observe that, at the Newton Branch Meeting of the Provincial Association, reported in the same number of the Journal, Mr. Burrows related the result of his experience of the above mode of treating fevers.]

He commences by clearing the primæ viæ. If the skin remained hot and dry, the mental faculties dull and cloudy, the limbs painful and weary, he ordered his patients to be stripped and enveloped in a sheet wrung out of cold water, and closely wrapped in thick blankets. This application was continued forty minutes, or more, according to the effect produced. During the interval warm diluents were freely administered, and when a copious perspiration ensued, the wrappings were removed, and the patient covered with the ordinary bedclothes. When the patient exhibited all the symptoms of "famine fever," viz: cold skin, feeble pulse, &c,, he modified the treatment by wringing the sheet out of very hot water, and covering the patient as before, at the same time gave hot negus and acetate of ammonia. When sweating was induced it was maintained by placing a hot brick wrapped in flannel at the feet.—The patients invariably expressed themselves relieved by this treatment, and some continued to convalesce from that period; others had a marked crisis on the eleventh to the fourteenth day. Mr. Burrows states that he feels convinced that, applied during the initiatory stage of fever, the wet sheet, with purgative and diaphoretic medicines, has prevented the further development of febrile action, and removed the first impression made by the poison upon the system.—*Ibid.*

